# Eye-Tracking-Driven Programming Metasurface System for Adaptive Beam Focusing and Polarization-Agile Communication

**DOI:** 10.34133/research.1225

**Published:** 2026-04-15

**Authors:** Shulei Zhang, Ruichao Zhu, Zuntian Chu, Chang Ding, Sai Sui, Sina Dang, Shaobo Qu, Jue Qu, Yuxiang Jia, Jiafu Wang

**Affiliations:** ^1^ Shaanxi Key Laboratory of Artificially-Structured Functional Materials and Devices, Air Force Engineering University, Xi’an, Shaanxi 710051, China.; ^2^ Air and Missile Defense College, Air Force Engineering University, Xi’an, Shaanxi 710051, China.

## Abstract

Expanding near-field regions in high-frequency 6G systems necessitates precise spatial-field control, yet narrow beamwidths and 3-dimensional tracking complexities create severe alignment hurdles. Here, we present an eye-tracking-driven programmable metasurface that bridges human visual intent with real-time electromagnetic responses via gaze-contingent beam steering. The system integrates a polarization-agile metasurface featuring independent 1-bit phase control for both copolarized and cross-polarized reflections. By engineering a 90° phase offset between these channels, the eye-tracking-driven programmable metasurface enables high-gain beam focusing and efficient linear-to-circular polarization conversion. Experimental results confirm that the system dynamically maps 3-dimensional gaze coordinates to metasurface coding patterns with millisecond-level responsiveness, facilitating robust near-field focusing and far-field scanning. This work establishes a “service-follows-vision” communication scenarios for intelligent wireless systems, offering distinct advantages in signal enhancement and interference mitigation.

## Introduction

Metasurfaces, capable of manipulating electromagnetic (EM) wave properties such as amplitude, phase, and polarization, have demonstrated transformative potential in beam deflection [1], absorption [2,3], focusing [4,5], and holography [6,7]. While static metasurfaces are constrained by fixed functionalities, the integration of tunable components has enabled programmable metasurfaces capable of real-time EM response reconfiguration [8,9]. The reconfiguration mechanisms have evolved from traditional electronic biasing [10] to more diverse stimuli, including optical pumping [11], thermal activation [12], and mechanical rotation [13]. Beyond wave manipulation, these agile responses allow metasurfaces to function as powerful platforms for information acquisition, facilitating high-sensitivity sensing, monitoring, and intelligent detection [14–16]. As a nearly passive and low-cost architecture, reconfigurable intelligent surfaces have emerged as a cornerstone for 6G wireless networks, offering a sustainable alternative to power-hungry active relay technologies [17,18].

The evolution toward millimeter-wave and terahertz bands in 6G systems has substantially expanded the near-field region, rendering the conventional far-field criterion (*R* > 2*D*^2^/*λ*) increasingly impractical [19]. Consequently, precise EM control within the near field has become a critical research focus [20]. Programmable metasurfaces are uniquely suited for near-field beam focusing, enabling simultaneous energy concentration in both angular and distance domains [21,22]. This precision not only minimizes signal leakage but also mitigates multipath effects, greatly enhancing communication efficiency [23]. However, the adoption of high-frequency bands necessitates increasingly narrow beamwidths, which escalates the technical difficulty of real-time beam alignment and target tracking.

To address these alignment challenges, various human-centric control paradigms—including brain wave analysis [24,25], gesture recognition [26,27], and computer vision [28]—have been explored to automate metasurface responses. Despite their potential, these modalities present distinct operational constraints: brain–computer interfaces are often hampered by hardware invasiveness and slow decoding speeds, while gesture-based systems occupy the user’s hands and require active manual effort. Similarly, computer vision-based tracking may fail to accurately capture a user’s immediate communication intent in multidevice environments. In contrast, eye-tracking technology naturally closes the loop between human intent and system response [29–31]. Since visual attention is intrinsically linked to psychological focus, gaze data provide a hands-free, real-time indicator of information consumption. This facilitates a “service-follows-vision” architecture, where high-gain beams are instantly directed to the user’s fixation point, offering a seamless high-speed experience in emerging augmented reality/extended reality scenarios [32,33].

Beyond beam focusing, polarization agility is paramount for mitigating cochannel interference and enhancing indoor communication security. Pioneering advancements of reconfigurable intelligent surfaces in linear and circular polarization control [34–37], 1-bit [38,39] to 2-bit [40–42] phase reconfigurability, dual-polarization support, and polarization conversion have laid critical foundations. However, in the realm of polarization agility, current approaches—whether relying on simultaneous amplitude–phase modulation [43–45] or employing a space-time coding strategy [46–48] to establish a complete orthogonal basis for synthesizing arbitrary polarization waves—demand high hardware complexity. These methods may face limitations when applied to large-scale arrays. A notable research gap remains in achieving adaptive, multimodal control that intelligently reconfigures both the polarization state and the focal point in response to dynamic user attention.

**Fig. 1.**
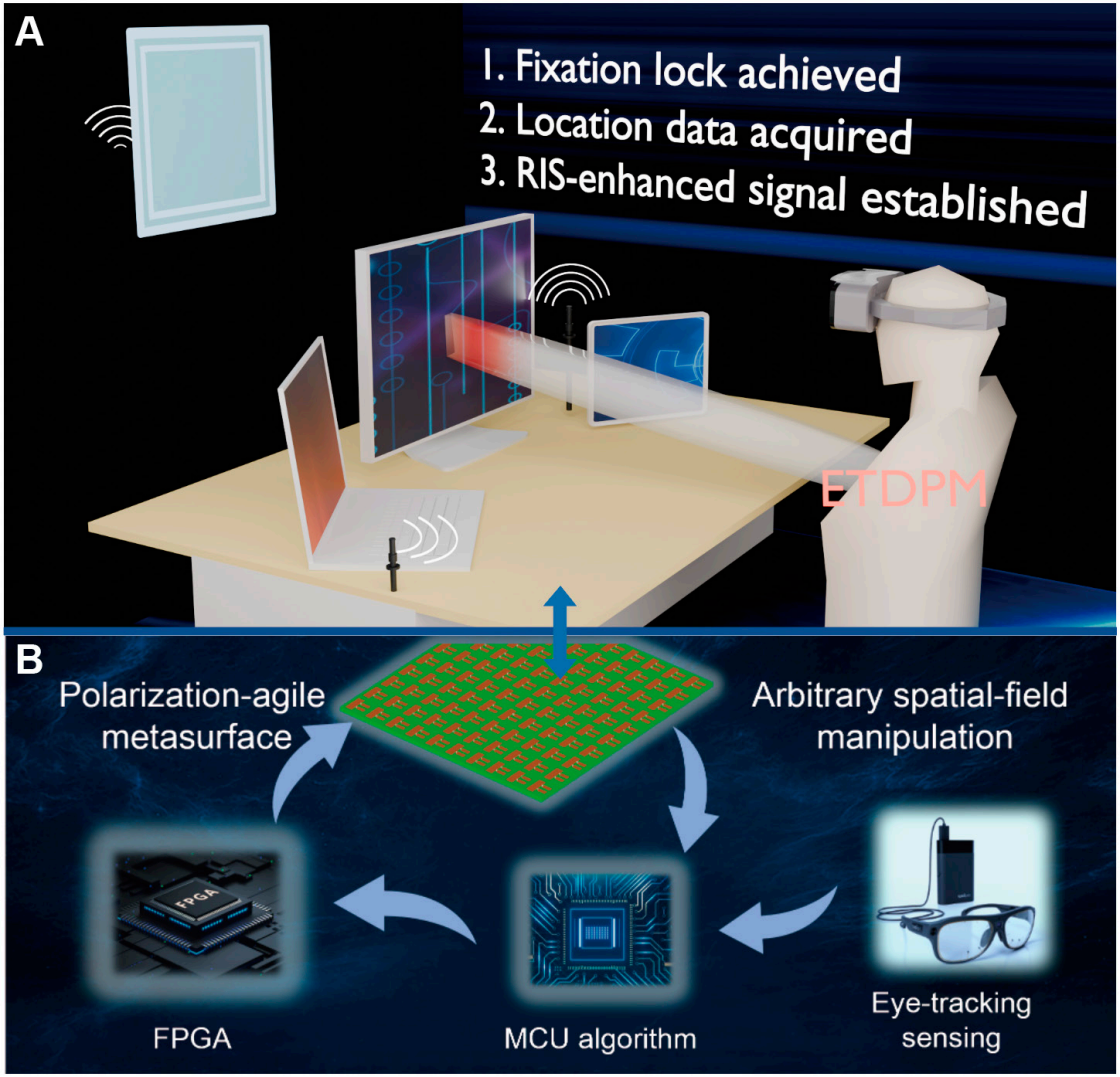
The framework of the proposed eye-tracking-driven programmable metasurface (ETDPM) system and its photographic scenes. The eye tracker identifies the user’s real-time focus location. The metasurface adjusts the phase compensation to concentrate electromagnetic energy at the detected fixation point. This framework enables wireless communication enhancement for targets in 2 polarization directions for indoor scenarios. (A) Scenario framework. (B) Closed-loop algorithm for metasurface systems.

In this work, we present an eye-tracking-driven programmable metasurface (ETDPM) system designed for real-time spatial-field beam focusing and polarization-adaptive communication. As illustrated in Fig. 1, the system architecture integrates augmented-reality-mounted eye trackers that capture ocular dynamics to drive EM responses. A central processor maps these 3-dimensional (3D) fixation coordinates to optimized 1-bit phase compensation patterns, which are then configured by a microcontroller in real time. This framework enables the metasurface to dynamically switch between vertical, horizontal, and circular polarization modes while maintaining high-gain focus at the user’s instantaneous visual target. To validate the system, we investigate 4 representative scenarios: (a) copolarized beam focusing, (b) cross-polarized beam focusing, (c) linear-to-circular polarization conversion, and (d) experimental wireless communication verification. Full-wave simulations and hardware characterizations confirm that the ETDPM exhibits millisecond-level reconfigurability and robust spatial-field signal enhancement. Systematic assessments further reveal its exceptional multipolarization multiplexing capabilities, establishing a human-centric paradigm for next-generation intelligent metasurface architectures.

## Results

### Meta-atom design

**Fig. 2.**
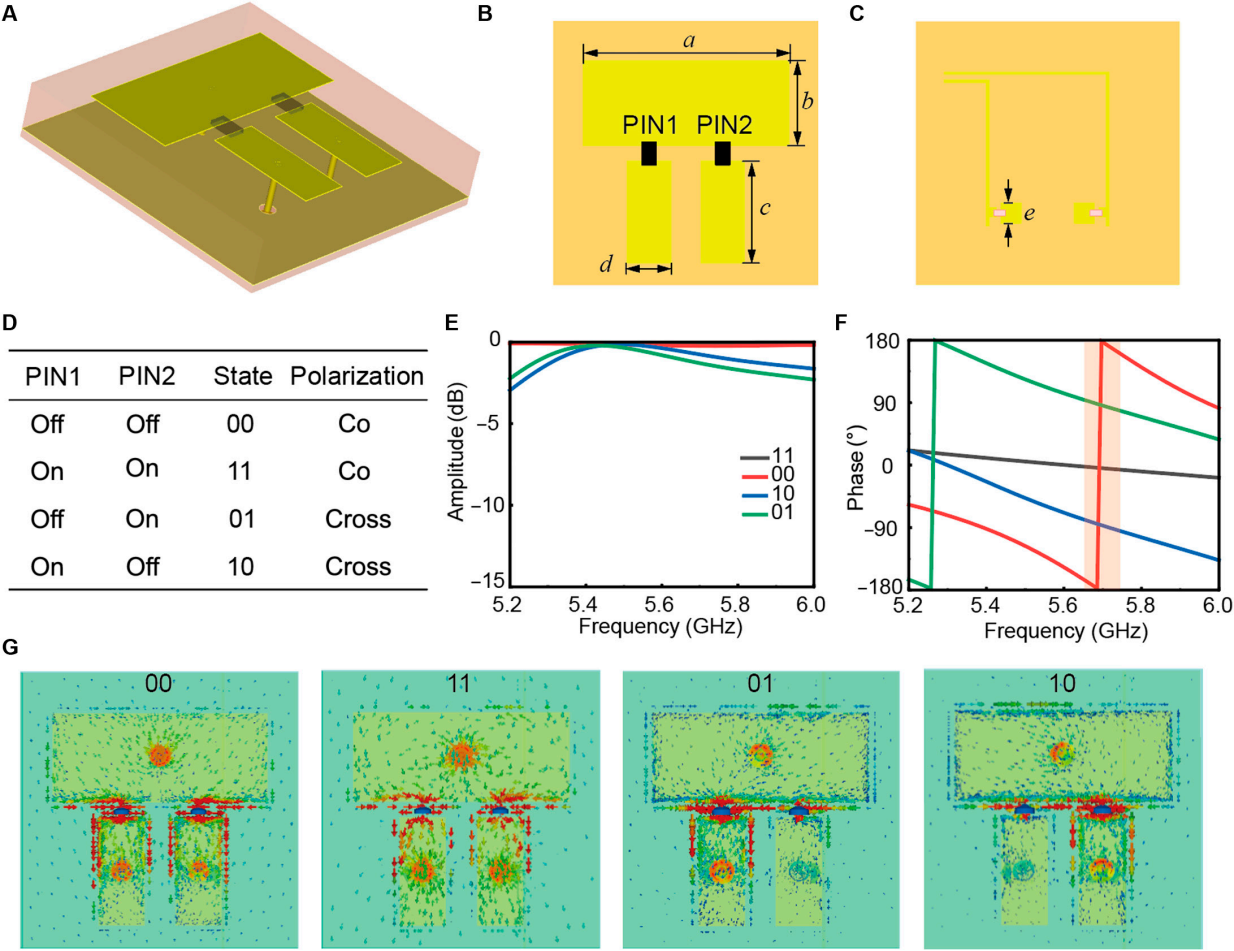
The eye-tracking-driven programmable metasurface (ETDPM) and its properties. The 3-dimensional (3D) view (A), top view (B), and bottom (C) view of the ETDPM. (D) Representation of diode switching states and polarization direction of reflected waves. The amplitude (E) and phase (F) under the 4 states, which achieve two 1-bit groups and allow linear-to-circular polarization conversion. (G) The current distribution of the meta-atom under the 4 states.

To achieve simultaneous control over both amplitude and phase for copolarized and cross-polarized reflections, we design a π-shaped structural unit. Figure 2A presents the 3D view of the proposed meta-atom, featuring 2 F4B dielectric layers (*ε_r_* = 2.65, tan *δ* = 0.01; *h*_1_ = 3 mm, and *h*_2_ = 0.5 mm) bonded by a 0.1-mm-thick prepreg. The trilayer metallic configuration consists of a top radiation layer, a ground layer, and a bottom bias layer. The top metal patch is connected to ground through blind vias for negative bias, and 2 smaller patches link to bottom-layer square patches via plated via holes for positive bias. As shown in Fig. 2C, the bottom square patch incorporates an 18-nH inductor with feed lines routed to external connectors. The geometrical parameters are *a* = 14 mm, *b* = 5.8 mm, *c* = 7 mm, *d* = 3 mm, and *e* = 1.4 mm, with unit cell periodicity *p* = 18 mm. Two surface-mounted PIN diodes (1320-079LF, PIN1, and PIN2) enable reconfigurability. The equivalent parameters of the PIN diode are *R* = 0.5 Ω and *L* = 0.75 nH in the on state and *L* = 0.5 nH and *C* = 0.24 pF in the off state. Under *y*-polarized wave incidence, the meta-atom exhibits 4 distinct operational states (Fig. 2D), which can be separated into 2 groups: (a) 00/11 states: Resonant current paths along the *y* direction generate *y*-polarized reflections. (b) 01/10 states: L-shaped current paths oriented at 45° relative to *x*/*y* axes induce polarization conversion, producing *x*-polarized reflections.

As shown in Fig. 2G, the L-shaped surface current paths of 01 and 10 are approximately tilted at 45°, forming a polarization-converting structure. Additionally, due to the opposite surface current directions of 01 and 10, these configurations should exhibit a reflection phase difference of 180°. By optimizing structural dimensions, a 180° phase difference between 00/11 states and between 01/10 states is achieved, meeting 1-bit phase control requirements for copolarization and cross-polarization reflection, respectively. Figure 2E shows the amplitude response under *y*-polarized excitation, demonstrating reflection magnitudes exceeding −3 dB across 5.3 to 5.8 GHz. States 00 and 11 exhibit a phase difference of approximately 180° around 5.7 GHz, while states 10 and 01 maintain a consistent 180° phase difference across the 5.3- to 5.8-GHz range. This provides 1-bit phase control for copolarized and cross-polarized reflection. Notably, we specifically optimize the structure to maintain a 90° phase offset between the 10/11 states. This quadrature relationship is the key to achieving efficient linear-to-circular polarization conversion.

### Beam focusing in the near field

For experimental validation of the proposed meta-atom’s beamforming performance, a 20 × 20-element programmable metasurface (ETDPM) is developed through full-cycle implementation including design, fabrication, and measurement. Initial simulation verification at 5.7 GHz involved beam-focusing control through coding sequence manipulation. The ideal phase distribution $\varphi_{\mathrm{mn}}$ for the element located in the *m*th row and *n*th column follows

$$\varphi_{\mathrm{mn}}=k_{0}\left(\left|{\hat{r}}_{\mathrm{mn}}-{\hat{r}}_{f}\right|-{\hat{r}}_{\mathrm{mn}}\cdot \hat{u}\right)+\varphi_{0}$$ (1)

where ${\hat{r}}_{\mathrm{mn}}$ is the element position vector, $k_{0}$ is the wave vector of the free space, ${\hat{r}}_{f}$ denotes the feed position vector, $\hat{u}$ indicates the unit vector of the desired reflected beam direction, $\varphi_{0}$ represents a constant reference phase, and *m* and *n* range from 1 to 20. This formulation stems from the duality between near-field feed reflection and far-field plane-wave focusing. For focal point positioning at $F=\left(x_{F},y_{F},z_{F}\right)$, the expression simplifies to

$$\varphi_{\mathrm{mn}}=\frac{2\pi }{\lambda }\left(\sqrt{{\left(x_{F}-x\right)}^{2}+{\left(y_{F}-y\right)}^{2}+z_{F}^{2}}\right)$$ (2)

where *x*, *y* denotes the spatial coordinates of the unit cell. Then, the continuous ideal phase distribution should be quantized as 2 states using the following criterion:

$$\varphi_{\mathrm{mn}}=\left\{\begin{array}{cc} 0{}^{\circ}, & 0{}^{\circ}<\varphi_{\mathrm{mn}}<180{}^{\circ}\\ 180^{{}^{\circ}}, & \text{otherwise} \end{array}\right.$$ (3)

**Fig. 3.**
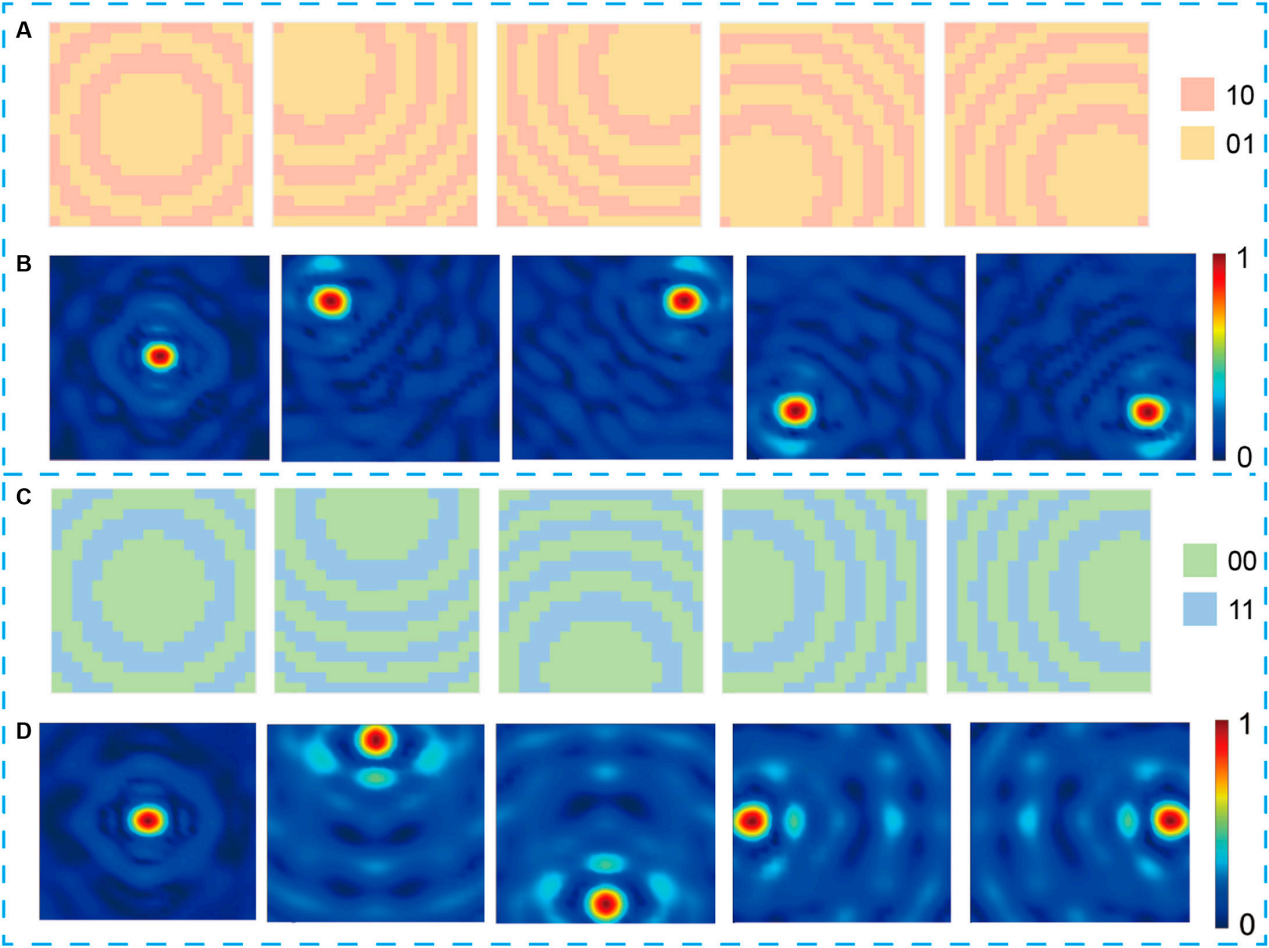
The phase distribution and the corresponding simulated results in the *xy* plane. The phase distribution (A) for focusing under cross-polarization reflection and the corresponding simulated cross-polarization results (B). The phase distribution (C) for focusing under copolarization reflection and the corresponding simulated copolarization results (D).

Therefore, the desired phase for focusing at the specified frequency can be calculated using the above equation, which corresponds to the switching state pattern of all diodes on the metasurface. CST simulations employed open boundary conditions, and a time-domain solver was utilized to calculate the electric field distribution within a specified spatial region. For cross-polarized reflection, the corresponding PIN diode states are 01 and 10. Five coordinate values are selected to demonstrate focusing at different locations: (0, 0, 150), (100, 100, 150), (−100, 100, 150), (−100, −100, 150), and (100, −100, 150). Figure 3A presents the quantized phase distribution maps corresponding to focusing at each of these 5 coordinate points. Cosimulation using MATLAB and CST can markedly simplify the layout generation process and enhance time efficiency. Figure 3B shows the simulated electric field distribution, clearly revealing high-intensity focusing of the electric field at the designated focal position.

As mentioned earlier, the unit exhibits copolarized reflection with a 180° phase difference between states 00/11 at 5.7 GHz. Similar to the method of cross-polarized reflection focusing discussed above, by applying the phases calculated from Eq. (3) to states 00 and 11, copolarized reflection focusing can be achieved. Another 5 coordinate values are selected to demonstrate focusing at different locations: (0, 0, 180), (0, 100, 180), (100, 0, 180), (−100, 0, 180), and (0, −100, 180). Compared to the cross-polarization focusing mentioned above, it not only changes the coordinate position on the 2-dimensional (2D) plane but also alters the depth of the focusing. Figure 3C and D present the quantized phase distribution maps for focusing at each of these 5 coordinate points, along with the simulated electric field distribution, which also proves the excellent results of copolarization reflection for single-point focusing. Additionally, as shown in Fig. 3, by centering on the focal point, the radius of the 3-dB focal intensity spot can be estimated to be approximately 2 cm. Given that the focal distance from the metasurface is 15 or 18 cm, the corresponding angular resolutions are arctan (2/15) ≈ 7.4° and arctan (2/18) ≈ 6.3°, respectively.

### Far-field and polarization conversion

**Fig. 4.**
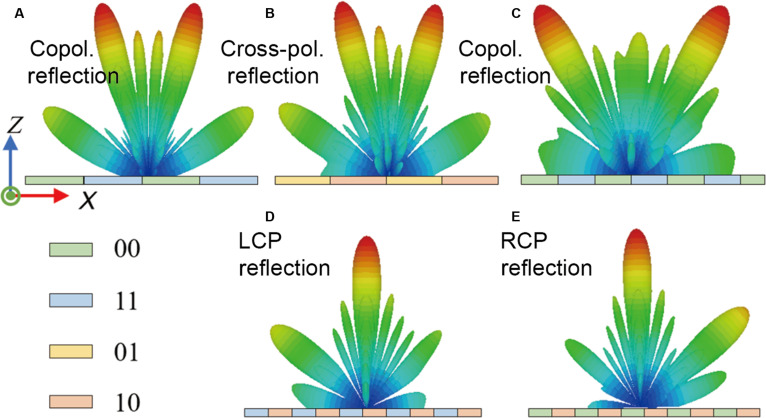
The simulated far-field radiation patterns in the *xz* plane (cross-sectional view) of beam deflection and polarization conversion under different state combinations. (A) Copolarization with the “AAAAABBBBB…” sequence, where A = 00 and B = 11. (B) Cross-polarization with the “AAAAABBBBB…” sequence, where A = 01 and B = 10. (C) Copolarization with the “AAABBB…” sequence, where A = 00 and B = 11. (D) *y*-polarization to left circular polarization (LCP) conversion using states 11/10. (E) *y*-polarization to right circular polarization (RCP) conversion using states 00/10.

When the observed object is at a relatively long distance or when high signal strength is not required, the far-field beam deflection effect can also be employed to enhance communication. A simplified Snell’s law was used to calculate dual-beam deflection, and the 2D far-field patterns under copolarized reflection and cross-polarized reflection with different periodicities were validated. Figure 4A to C illustrate simulation results of beam deflection angles for 2 periodic arrangements: the sequences “AAAAABBBBB” and “AAABBB”, with corresponding periods of 90 and 54 mm, where A and B represent 1 of the 4 states (00, 11, 01, and 10, identical in the *y* direction). The formula predicts these deflection angles to be 18° and 28° at 5.7 GHz, respectively.

The phase relationship ($\Delta \phi =90^{{}^{\circ}}$) among the operational states enables efficient linear-to-circular polarization transformation. Under y-polarized wave incidence, circular polarization is synthesized by engineering the precise phase offset between the orthogonal reflected components. As illustrated in Fig. 2F, copolarized states 11 and 00 are optimized to provide a 90° phase lead and lag relative to the cross-polarized state 10, respectively. Consequently, left circular polarization (LCP) is generated through the +90° phase advancement of the *y* component (utilizing states 11 and 10), while right circular polarization (RCP) emerges from the corresponding −90° retardation (utilizing states 00 and 10). This phase-controlled handedness is experimentally validated by the high-fidelity far-field scattering results presented in Fig. 4D and E.

To mitigate performance degradation at extended focal distances, the ETDPM can be reconfigured to operate in a high-directivity beam-steering mode. In this configuration, a horn antenna is positioned 360 mm from the metasurface center (*F*/*D* = 1) to serve as the feed source, as shown in Fig. S6. By applying phase compensation algorithms to generate the required linear phase gradients, the system achieves wide-angle beam scanning across both polarization states.

**Fig. 5.**
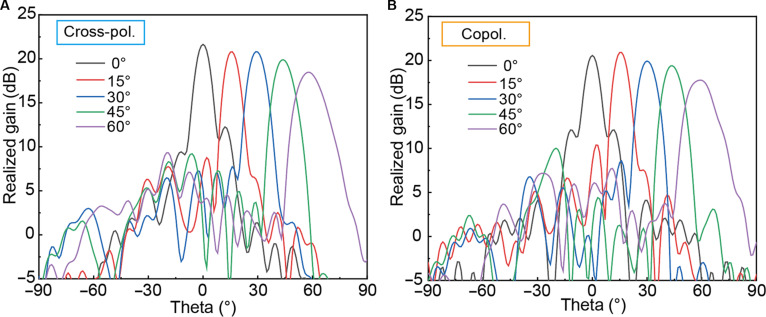
Simulated radiation patterns of the proposed eye-tracking-driven programmable metasurface (ETDPM) for different scan angles and polarization. (A) Cross-polarization. (B) Copolarization.

As illustrated in Fig. 5, the simulated radiation patterns confirm that the metasurface maintains high realized gains of approximately 20 dB for both cross-polarization and copolarization modes (Fig. 5A and B). The scanning range extends up to 60° with a minimal scan loss of only 3.8 dB. Quantitatively, the simulated half-power beamwidths are measured at 9.8° for cross-polarization and 10.3° for copolarization. Furthermore, the side-lobe levels consistently remain below −12 dB, demonstrating excellent beam collimation and efficient energy concentration. These results validate the system’s operational efficacy in transitioning from near-field focusing to far-field steering for robust spatial-field communication.

### Fixation position extraction via an eye tracker

Eye-tracking technology typically employs the pupil-corneal reflection method, which locates fixation positions by emitting infrared light and processing images of the eye illuminated by the infrared light. In this study, Tobii Pro Glasses 3 is used to collect users’ eye movement data. Glasses 3 can record real-time saccadic trajectories and fixation positions, with built-in robust data processing capabilities that enable easy acquisition of 2D *x*/*y* coordinates for fixation points. In indoor virtual reality scenarios, objects are usually fixed in position, allowing the determination of 3D coordinates of the fixated object once the 2D coordinates are obtained. After the computer receives the 3D coordinates, they are mapped to the metasurface’s intrinsic coordinate system to establish positional information for the focal point. The metasurface further supports reflections using different polarized waves to achieve higher anti-interference performance. The calculated phase of metasurface is sent to an Arduino, which then dynamically controls the switching states of PIN diodes in real time to achieve the desired beam control.

**Fig. 6.**
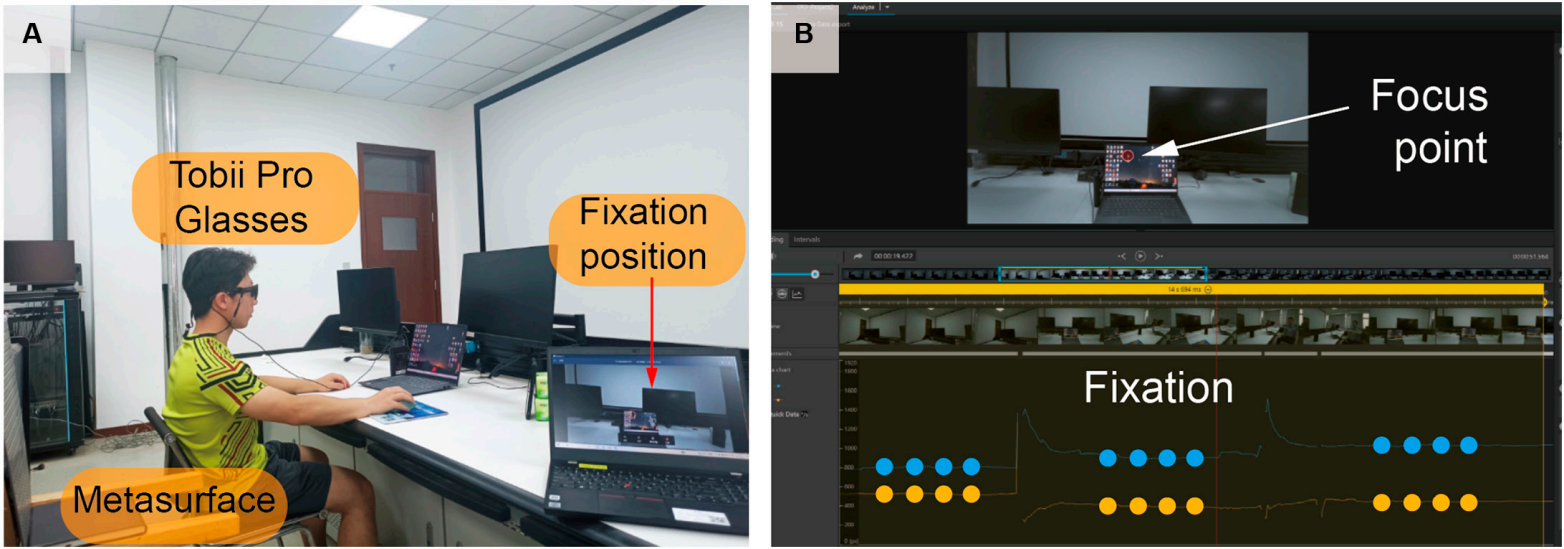
Eye-tracking test. Scene diagram showing measured gaze locations (A) and fixation tracking process images processed by Tobii Lab (B).

The experiment tested Glasses 3’s ability to acquire fixation positions in a practical scenario. Participants sequentially fixated on 3 objects in front of them: a door, a laptop, and a smartphone. Tobii Glasses 3 captures real-time fixation positions, represented as red circles on the screen, and the data are subsequently extracted and analyzed to obtain the *x*/*y* coordinates of the fixation points. Figure 6A delineates the experimental setup for fixation tracking measurement, where the right-side laptop monitors fixation positional data in real time, visualized as red circular markers superimposed on the visual stimulus interface. Figure 6B presents the temporal–spatial distribution of fixation–saccade patterns, exhibiting 3 approximately linear trajectory segments with prolonged durations. These quasi-stationary phases correspond to 3 distinct visual attention foci: the door panel, laptop interface, and mobile device, sequentially aligned with the predefined regions of interest. Partial data of the positional information for the fixation points are listed in Table S1. Three highlighted fixation point coordinates can be observed, corresponding to the aforementioned 3 objects. Real-time gaze position information can be obtained using the official Tobii Pro software development kit and sent to MATLAB to drive a field-programmable gate array for custom beamforming. Comprehensive video documentation and supplementary photographic records are archived in the Supplementary Materials.

Spatial mapping is established through initial calibration and real-time synchronization with the eye tracker’s built-in inertial measurement unit (recording pitch, yaw, and roll) to dynamically compensate for user head movements. Robustness is ensured by an alignment margin where the metasurface’s beamwidth (>6.3°) considerably exceeds the eye tracker’s precision (<1°), ensuring high-precision coverage and stable links despite minor mapping discrepancies. The end-to-end system latency is estimated at 30 to 50 ms, encompassing eye-tracking sensing (10 to 20 ms), computer-based coordinate mapping (<10 ms), and hardware control distribution (~10 ms). This response time is markedly shorter than the average human fixation duration of 200 to 300 ms, ensuring seamless real-time tracking of visual attention without perceptible lag. Combined with the nanosecond-scale switching of PIN diodes, the ETDPM framework provides the high-speed reconfiguration necessary for robust, intent-driven communication.

### Fabrication and experimental setup of the metasurface

The metasurface sample, fabricated via printed circuit board technology, consists of a 20 × 20 array of unit cells with a total area of 380 × 380 mm. The top (a) and bottom (b) of the fabricated sample and the control circuit board are illustrated in Fig. S2. There are 2 PIN diodes on each meta-atom, so in order to control the states of 800 diodes, the bottom layer incorporates 800 feed lines. Every 20 feed lines are soldered to a 20-pin connector, which is then interfaced via flexible printed circuit cables to the control board. The control board comprises 50 cascaded shift registers, enabling serial input and parallel output with latch functionality. This design allows parallel control of all diodes’ switching states using minimal input/output ports, specifically the serial data, storage clock pulse, shift clock pulse, and output enable pins. The schematic of the control circuit board is shown in Fig. S3. Any desired beamforming effects can be achieved by converting the MATLAB-computed phase matrices into 8-bit arrays and sequentially inputting them into cascaded shift registers. The system was calibrated using a metal plate of identical dimensions to the sample. We measured the amplitude and phase responses under both copolarized (states 00 and 11) and cross-polarized (states 10 and 01) reflection conditions. As shown in Fig. S4, the experimental results exhibit close agreement with simulations in fundamental characteristics while demonstrating a frequency shift of approximately 0.2 GHz, which is attributed to fabrication errors.

**Fig. 7.**
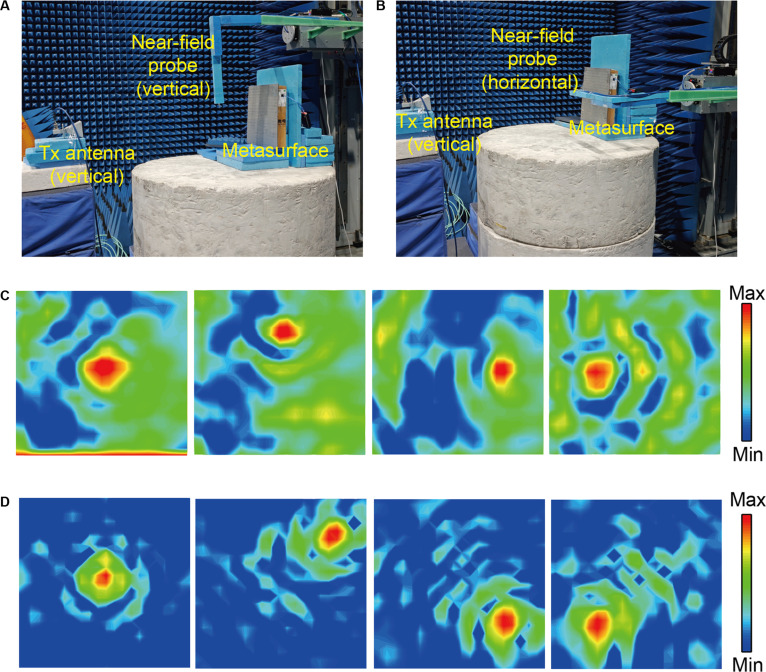
Near-field testing imaging and analytical results. Copolarization near-field probe energy scanning (A) and the experimental results (C). Cross-polarization near-field probe energy scanning (B) and the experimental results (D).

The planar near-field scanning technique was employed to characterize the electric field distribution in the focal region over a scan area of 400 mm × 400 mm to cover the prototype. Two polarization scenarios were measured separately, with both probes placed at a specified distance in front of the metasurface according to the predetermined positions. As shown in Fig. 7, a vertically polarized horn antenna served as the feed source, positioned 1.5 m from the vertically oriented metasurface to ensure plane-wave illumination. Figure 7A and B depict the experimental setups with vertically and horizontally oriented near-field probes, respectively, used to evaluate copolarized (*y*-pol) and cross-polarized (*x*-pol) reflection focusing effects. Figure 7C and D demonstrate the measured *x*-pol and *y*-pol focusing results. The experimental data align well with simulations, with focal points near predefined positions. Notably, copol measurements exhibit slightly higher background field levels due to feed interference, while *x*-pol results show superior focusing purity owing to minimal antenna influence.

**Fig. 8.**
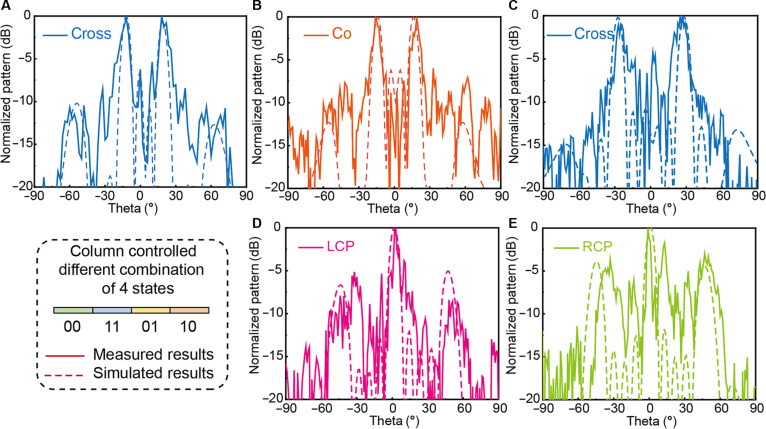
The measured 2-dimensional (2D) far-field scattering results at 5.9 GHz under *y*-polarized wave incidence (A) Cross-polarization with the “AAAAABBBBB…” sequence, where A = 01 and B = 10. (B) Copolarization with the “AAAAABBBBB…” sequence, where A = 00 and B = 11. (C) Cross-polarization with the “AAABBB…” sequence, where A = 01 and B = 10. (D) *y*-polarization to left circular polarization (LCP) conversion using states 11/10. (E) *y*-polarization to right circular polarization (RCP) conversion using states 00/10.

Linearly and circularly polarized horn antennas were used as receivers. The metasurface and transmit horn remained stationary, while the receive horn was rotated on a turntable to acquire 2D far-field scattering patterns. Both antennas were placed over 1.5 m from the metasurface to satisfy plane-wave conditions. Figure 8A and B illustrate dual-beam deflection at 18° for *x*-pol and *y*-pol reflections, respectively, while Fig. 8C shows a 28° cross-polarization beam deflection. Figure 8D and E present LCP and RCP beams synthesized from *y*- and *x*-polarized waves with a 90° phase difference, respectively, demonstrating excellent agreement with simulations.

In our prototype, the control board is powered by a 5-V supply. To ensure robust switching and visual indication, the current limiting resistor was set to drive each PIN diode at approximately 10 mA. Consequently, the power consumption drawn from the supply for each active channel is $P_{\text{unit}}=V_{\mathrm{CC}}\times I=5\;V\times 10\;\mathrm{mA}=50\;\mathrm{mW}$. With an average 50% duty cycle for the 800-element array (400 active channels), the total power consumption is $P_{\text{total}}=400\times 50\;\mathrm{mW}=20\;W$. In fact, for the low-current SMP1320-079LF, power-hungry diagnostic light-emitting diodes and buck resistors are unnecessary. Thus, it requires only 0.7 V to turn on, with a resulting power consumption of merely 2.8 W.

### Verification of wireless communication

**Fig. 9.**
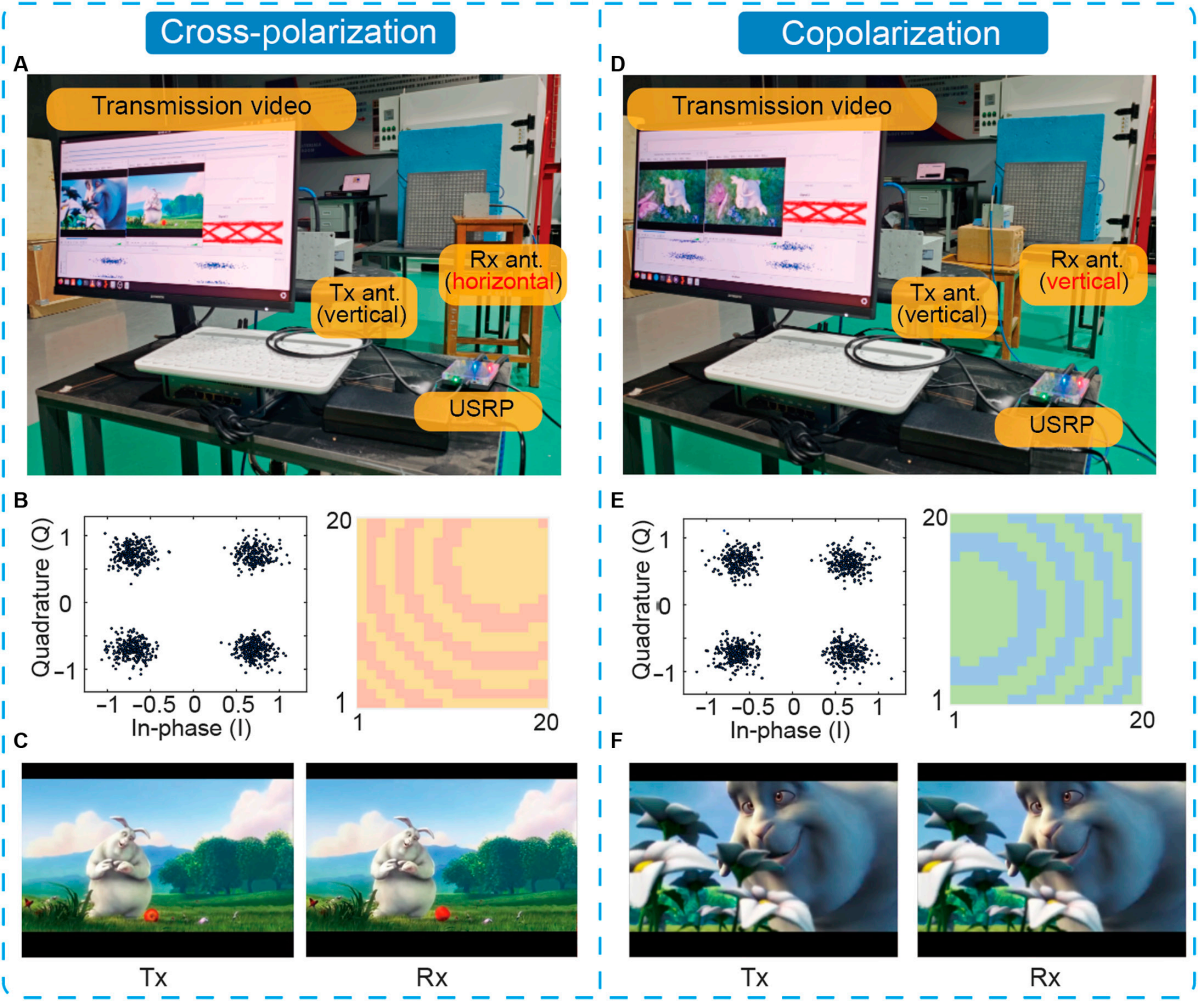
Communication test photos and results. (A to C) Real-time wireless video transmission photos under cross-polarization (A), corresponding constellation diagrams/phase distributions (B), and transmitted/received video screenshots (C). (D to F) Real-time wireless video transmission photos under copolarization (D), corresponding constellation diagrams/phase distributions (E), and transmitted/received video screenshots (F).

To validate the communication potential of the proposed polarization-reconfigurable metasurface controlled by an eye tracker, we established a wireless communication system to test its focusing capabilities in indoor scenarios. The system is mainly composed of a computer, a universal software radio peripheral, a pair of horn antennas, and the prototype. The software platform of this system operates on the Linux operating system, while the hardware platform implements a software-defined radio built upon the Artix-7 100T field-programmable gate array, which integrates an AD9361 dual-channel radio-frequency transceiver chip. Leveraging this integrated software–hardware architecture, the system achieves real-time video transmission capabilities. As illustrated in Fig. 9, the metasurface is vertically positioned, with a transmitting horn antenna (vertically polarized) placed 1 m directly in front of it. The receiving antenna was adjusted based on different focal positions to evaluate communication performance. Video data were converted into bitstreams, modulated using quadrature phase shift keying (QPSK), and transmitted via the ETDPM. Both copolarized and cross-polarized reflection focusing were tested, with the polarization orientation of the receiving horn antenna (vertical or horizontal) adjusted through rotation. Figure 9A demonstrates real-time video transmission performance at the focus point (100, 100, 150), while Fig. 9D shows transmission results at the focus point (−100, 0, 180). Figure S7 presents transmission effects at nonfocused positions.

Constellation diagrams during the transmission process are displayed in Fig. 9B and E, while Fig. 9C and F show screenshots of transmitted and received video frames. Comparative analysis reveals superior transmission quality at focused positions, whereas nonfocused positions exhibited issues such as video freezing and transmission interruptions, consistent with near-field experimental observations. Enhanced signal energy in designated directions ensures communication speed and data integrity, effectively verifying the system’s exceptional beam-focusing capabilities.

To enhance link robustness, a closed-loop heuristic strategy is integrated into the ETDPM system. As illustrated in Fig. S8, when the signal-to-noise ratio falls below a predefined threshold, a 1-bit feedback signal triggers the metasurface to rapidly switch among 4 precalibrated polarization modes—copol, cross-pol, LCP, and RCP—to restore signal quality. This adaptive mechanism maintains focused beam coordinates while ensuring stable communication in dynamic or interference-prone environments.

## Discussion and Conclusion

This study presented an ETDPM system designed for real-time beam focusing and polarization-adaptive communication. The system architecture establishes a direct mapping between human gaze dynamics and EM responses. The 4 states of the metasurface can achieve 4 polarization waves with great beam steering or focus accuracy. Numerical simulations and experimental characterizations at 5.9 GHz verified the system’s multimode capabilities, including precise near-field focusing and wide-angle far-field beam steering up to 60°. The integrated system demonstrates a total end-to-end latency of 30 to 50 ms, which is considerably lower than the average human fixation duration, ensuring real-time alignment. The system achieves high-precision gaze-to-spatial mapping with an eye-tracking accuracy of less than 1°, which is well within the 3-dB beamwidth (>6.3°) of the metasurface’s scanning beams, ensuring robust link coverage even with minor user movements. Furthermore, the implementation of a heuristic polarization selection strategy based on signal-to-noise ratio feedback allows the system to autonomously adapt to interference-prone environments. Wireless video transmission experiments confirmed stable high-quality links at focused positions, validating the operational efficacy of the ETDPM in practical “service-follows-vision” communication scenarios.

## Data Availability

The data that support the findings of this study are available from the corresponding authors.
