# Supplementary material for: Eye-Tracking-Driven Programming Metasurface System for Adaptive Beam Focusing and Polarization-Agile Communication
Source: Research (Wash D C). 2026 Apr 15;9:1225. doi: 10.34133/research.1225 (PMC13080098; doi:10.34133/research.1225)
Supplement: Supplementary 1 — Supplementary Text Figs. S1 to S8 Table S1 [file research.1225.f1.docx]

Supplementary Information

**Eye-Tracking-Driven Programming Metasurface System for Adaptive Beam Focusing and Polarization-agile Communication**

Shulei Zhang, Ruichao Zhu*, Zuntian Chu*, Chang Ding, Sai Sui, Sina Dang*, Shaobo Qu, Jue Qu, Yuxiang Jia, and Jiafu Wang*

Shulei Zhang, Ruichao Zhu, Chang Ding, Sai Sui, Shaobo Qu, Yuxiang Jia, Jiafu Wang

1 Shaanxi Key Laboratory of Artificially-Structured Functional Materials and Devices, Air Force Engineering University, Xi'an, Shaanxi 710051, China

E-mail: [zhuruichao1996@163.com](mailto:zhuruichao1996@163.com), [wangjiafu1981@126.com](mailto:wangjiafu1981@126.com)

Zuntian Chu, Sina Dang, Jue Qu

2 Air and Missile Defense College, Air Force Engineering University, Xi’an, Shaanxi 710051, China

E-mail: [15529381518@163.com](mailto:15529381518@163.com) , [dsnsw123@126.com](mailto:dsnsw123@126.com)

1. **Polarization-adaptive metasurface**

**
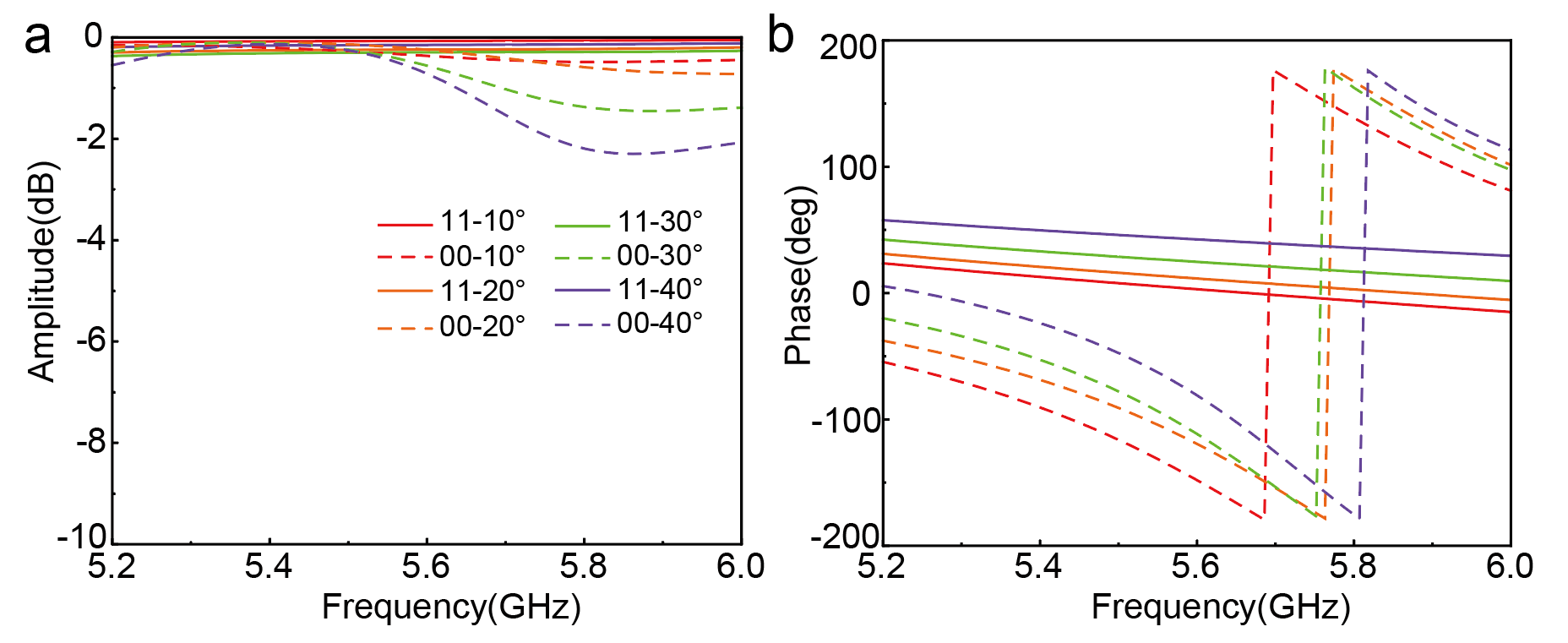
**

Figure S1 Co-polarization performance under obique incidence.


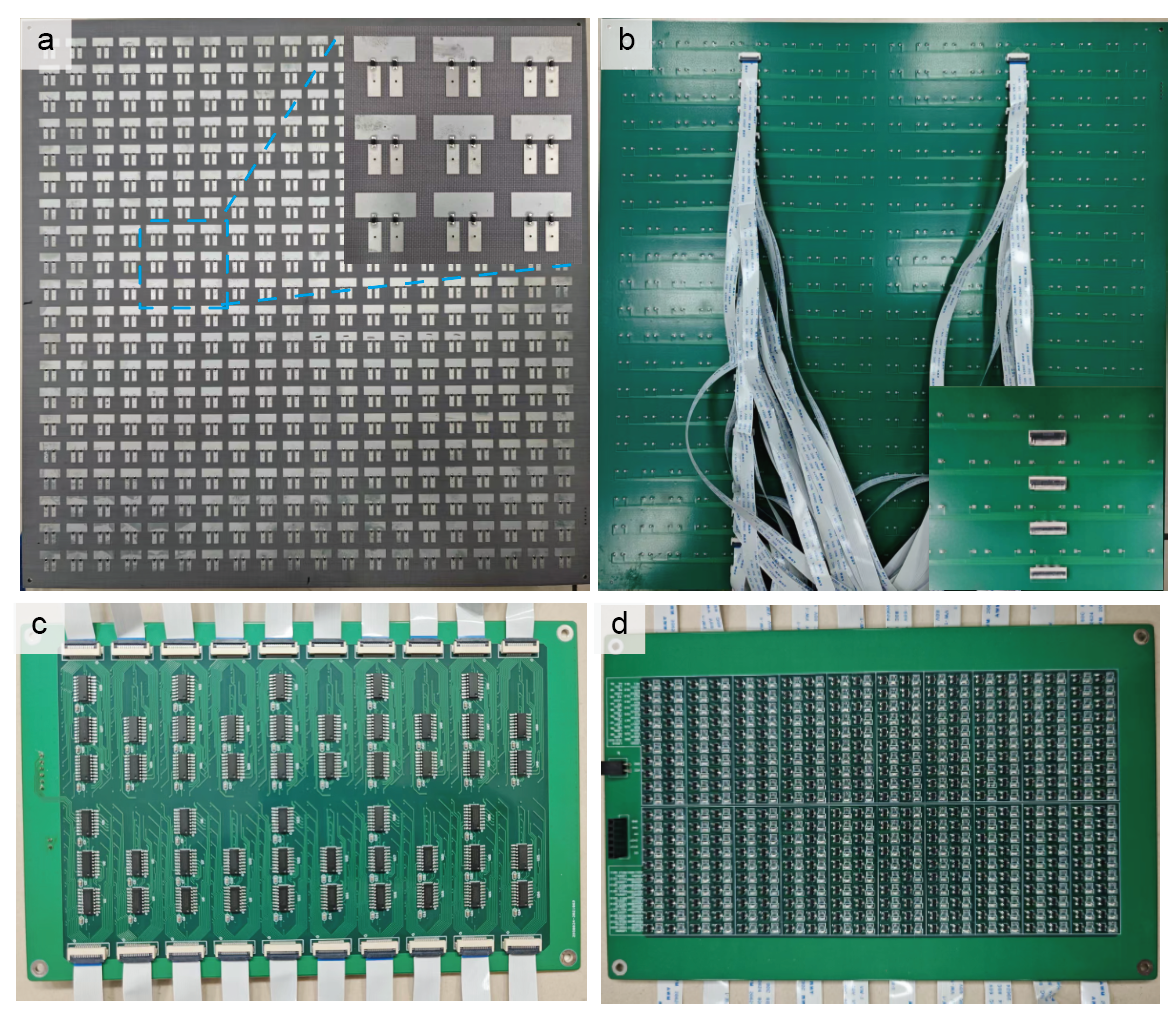


Figure S2 Top (a) and bottom (b) of the fabricated sample, and top (c) and back (d) of the control circuit board.


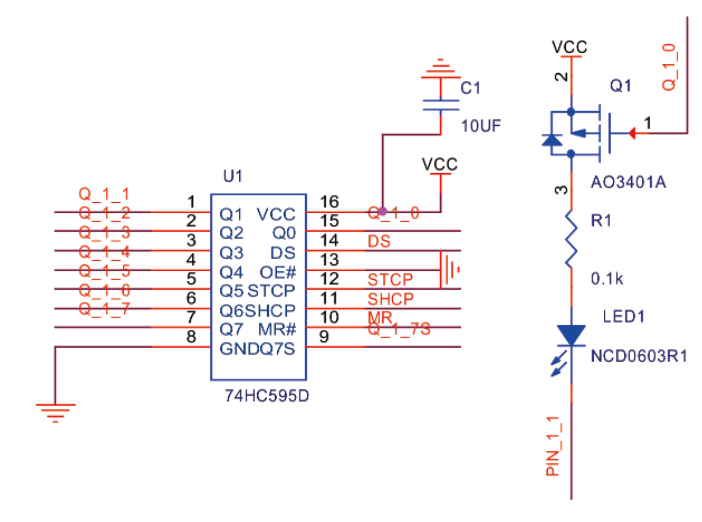


Figure S3 Schematic of the control circuit board.

As shown in Figure S4, the measured amplitude and phase responses are generally consistent with simulation results, with a slight frequency shift of approximately 0.5 GHz towards higher frequencies. Near 6.2 GHz, the amplitude for all four states remains above -3 dB. Furthermore, the phase difference between states ‘00’/‘11’ is 180°, and the phase difference between states ‘01’/‘10’ is also 180°, with these pairs exhibiting 90° phase separation.

Nevertheless, upon performing the actual near-field and far-field tests, it was observed that the device exhibited good performance within the operating frequency band of roughly 5.8-6.1GHz. This phenomenon could potentially be attributed to the strong coupling among the meta-atoms, leading to a frequency offset.


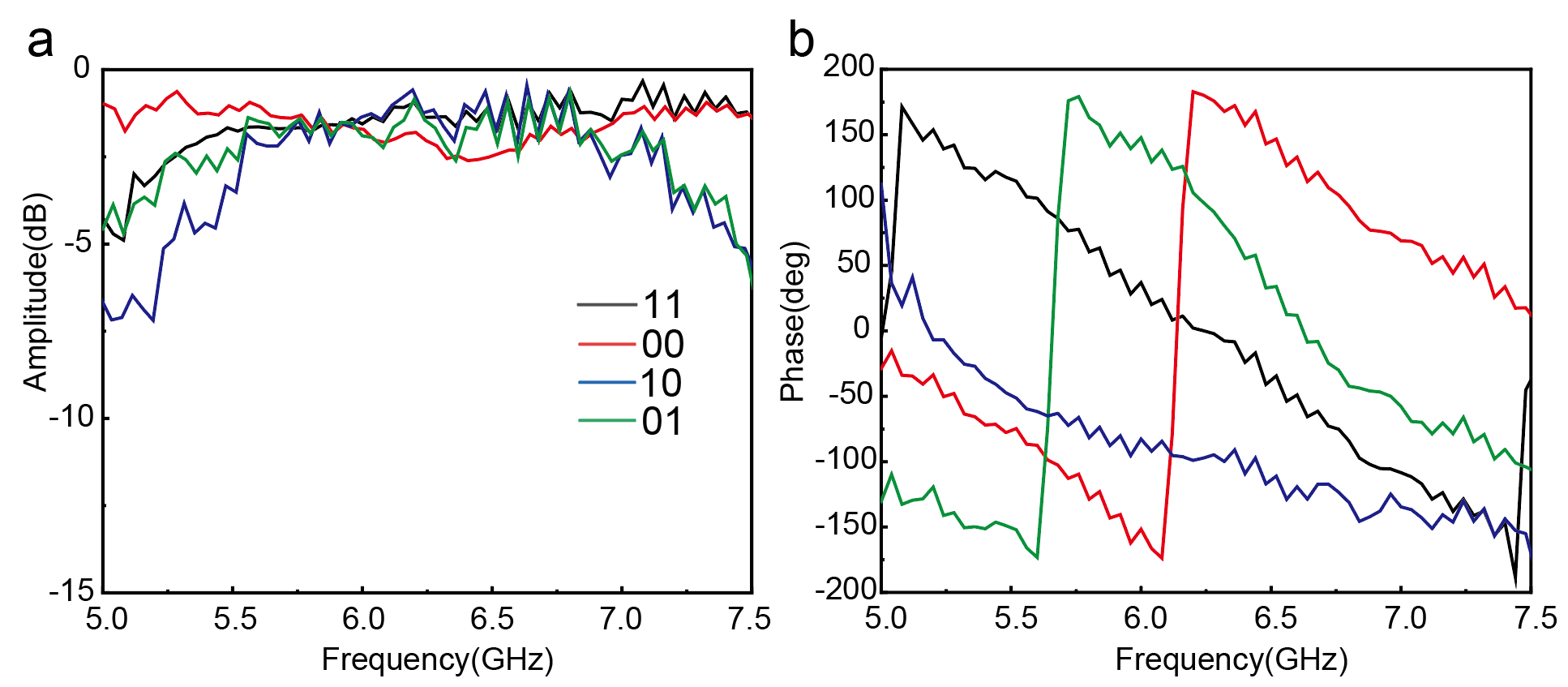


Figure S4 The measured amplitude and phase responses of the four states (00, 11, 01 and 10).

1. **Eye-Tracker and Eye-Movement Data**

The Tobii Pro Glasses 3 is an advanced eye-tracking device designed for naturalistic research, boasting a sampling rate of 240 Hz for single-eye tracking and 120 Hz for dual-eye tracking, ensuring the capture of dynamic eye movements with exceptional detail. Its accuracy typically ranges from 0.4° to 1.0°, complemented by a precision of 0.1° to 0.2°, providing reliable data even in diverse real-world environments. With a latency of under 10 ms, it enables precise synchronization of fixation data with external stimuli and user actions. Featuring two high-resolution eye cameras and a 1080p scene camera for capturing the user’s visual field, the device also incorporates an integrated microphone for simultaneous audio recording.

The testing procedure requires the wearer to first complete a simple eye position calibration. Table S1 details the eye-tracking monitoring process and its corresponding fixation data, providing access to information such as event type, duration, and fixation position coordinates. As depicted in Video S1, the eye tracker enables continuous monitoring of fixation position. The computer subsequently receives this data and transmits it to the metasurface for controlled signal enhancement at the respective positions.


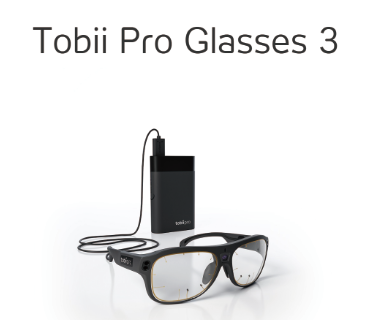


Figure S5 Image of the Tobii Pro Glasses 3.

Table S1: Eye-tracking monitoring data and coordinates of fixation points(partial).

| Interval | Event_type | Validity | Event- Index | Start | Stop | Duration | Fixation  PointX | Fixation  PointY |
| --- | --- | --- | --- | --- | --- | --- | --- | --- |
| 1 | Fixation | Whole | 23 | 10560 | 11081 | 521 | 0.4018 | 0.46195 |
| 1 | Saccade | Whole | 31 | 11081 | 11101 | 20 |  |  |
| 1 | Fixation | Whole | 24 | 11101 | 15268 | 4168 | 0.41547 | 0.51066 |
| 1 | Saccade | Partial | 32 | 15268 | 15288 | 20 |  |  |
| 1 | Fixation | Partial | 25 | 15389 | 15509 | 120 | 0.71147 | 0.75195 |
| 1 | Saccade | Whole | 33 | 15509 | 15529 | 20 |  |  |
| 1 | Fixation | Whole | 26 | 15529 | 16591 | 1062 | 0.53563 | 0.63179 |
| 1 | Saccade | Whole | 34 | 16591 | 16611 | 20 |  |  |
| 1 | Fixation | Whole | 27 | 16611 | 19476 | 2865 | 0.47541 | 0.6303 |
| 1 | Saccade | Whole | 35 | 19476 | 19496 | 20 |  |  |
| 1 | Fixation | Whole | 28 | 19496 | 20318 | 821 | 0.4946 | 0.64745 |
| 1 | Saccade | Partial | 36 | 20318 | 20338 | 20 |  |  |
| 1 | Fixation | Partial | 29 | 20418 | 20478 | 60 | 0.72543 | 0.72553 |
| 1 | Saccade | Partial | 37 | 20478 | 20498 | 20 |  |  |
| 1 | Saccade | Partial | 38 | 20538 | 20558 | 20 |  |  |
| 1 | Fixation | Whole | 30 | 20558 | 21500 | 942 | 0.56251 | 0.60026 |
| 1 | Saccade | Partial | 39 | 21500 | 21520 | 20 |  |  |
| 1 | Saccade | Partial | 40 | 21600 | 21680 | 80 |  |  |
| 1 | Fixation | Whole | 31 | 21680 | 27250 | 5570 | 0.53653 | 0.58379 |
| 1 | Saccade | Partial | 41 | 27250 | 27290 | 40 |  |  |
| 1 | Saccade | Partial | 42 | 27330 | 27390 | 60 |  |  |
| 1 | Fixation | Whole | 32 | 27390 | 27471 | 80 | 0.79545 | 0.6063 |

1. **Farfield beam steering performance**


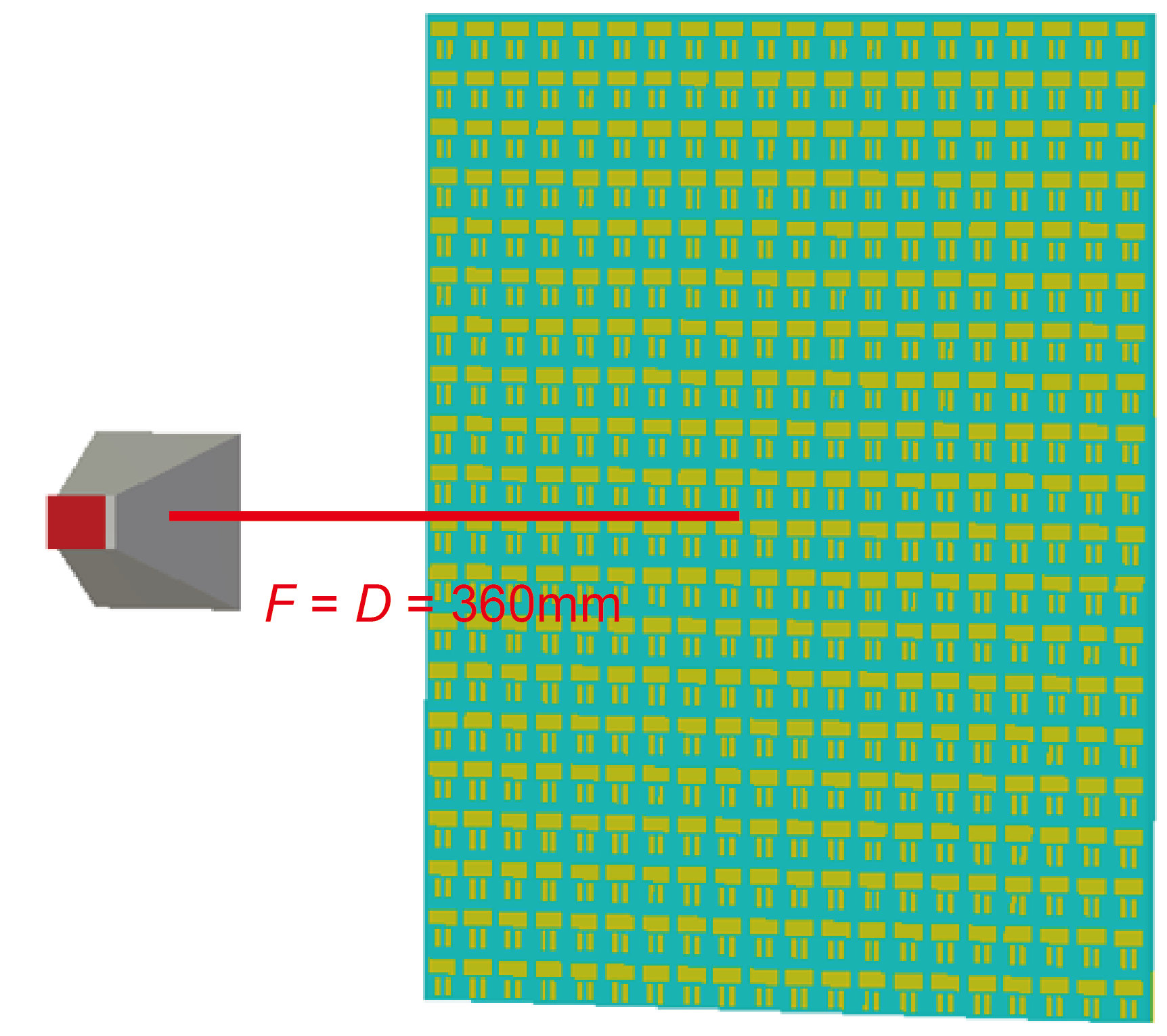


Figure S6 Simulation set-up.

1. **Communication at Non-Focused Positions**

Using the same phase distribution as in Figure 8 (c), the desired focal point should be at upper-right (100,100,150). To evaluate wireless communication performance at non-focal positions, the receiver horn antenna was placed at a left-side non-focal position (Figure S4), where the distorted constellation diagram indicates failed video decoding.


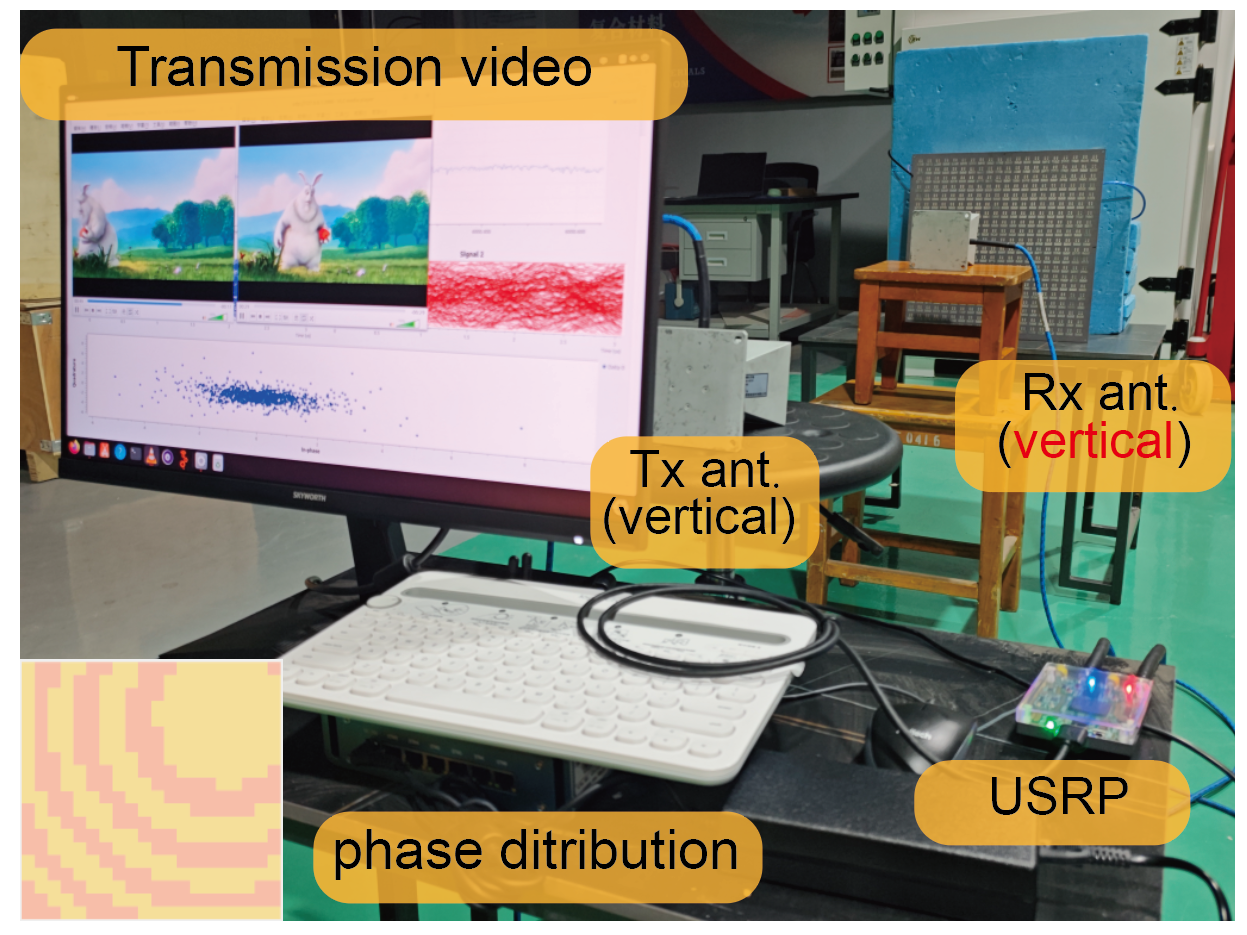


Figure S7 Photograph of the wireless communication test at a non-focused position.

1. **The systemic framework**


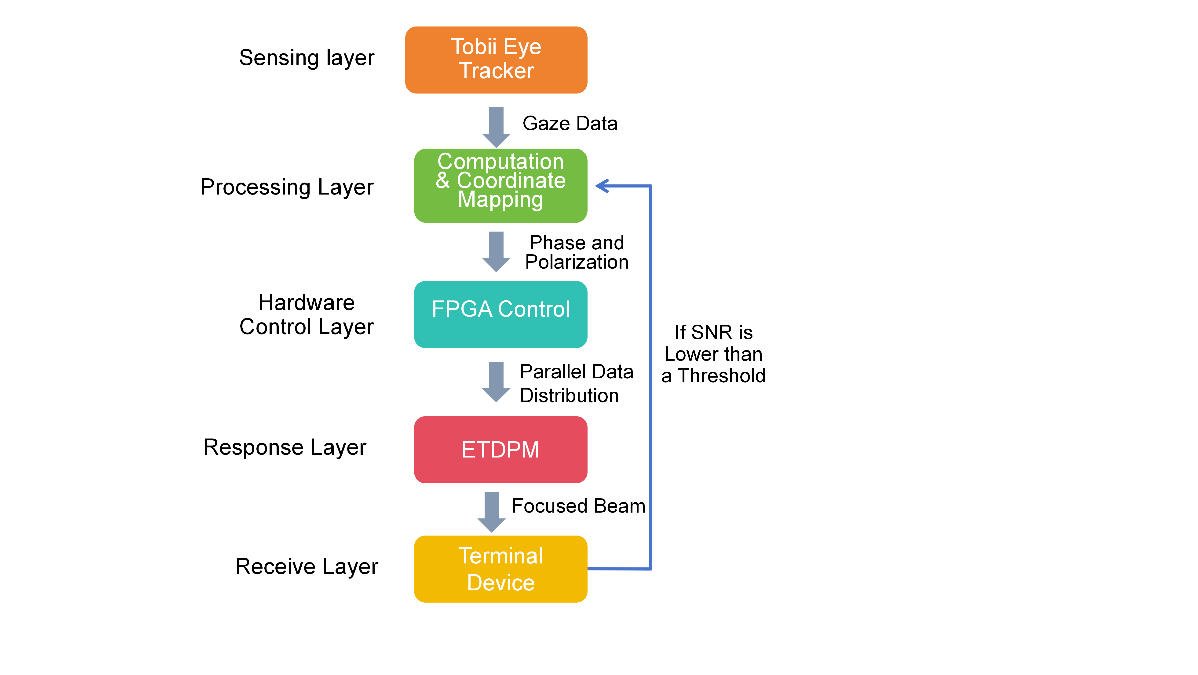


Figure S8 The systemic framework and the operational logic of the ETDPM system.
